# Simulation and Experiment of the Trapping Trajectory for Janus Particles in Linearly Polarized Optical Traps

**DOI:** 10.3390/mi13040608

**Published:** 2022-04-13

**Authors:** Xiaoqing Gao, Cong Zhai, Zuzeng Lin, Yulu Chen, Hongbin Li, Chunguang Hu

**Affiliations:** 1 State Key Laboratory of Precision Measuring Technology and Instruments, School of Precision Instrument and Optoelectronics Engineering, Tianjin University, Tianjin 300072, China; gxq2009@tju.edu.cn (X.G.); oliverchild@tju.edu.cn (C.Z.); linzuzeng@tju.edu.cn (Z.L.); tjucyl@tju.edu.cn (Y.C.); 2 Department of Chemistry, University of British Columbia, Vancouver, BC V6T 1Z1, Canada; hongbin@chem.ubc.ca

**Keywords:** optical trapping, controllable rotation, Janus particles, *T*-matrix method

## Abstract

The highly focused laser beam is capable of confining micro-sized particle in its focus. This is widely known as optical trapping. The Janus particle is composed of two hemispheres with different refractive indexes. In a linearly polarized optical trap, the Janus particle tends to align itself to an orientation where the interface of the two hemispheres is parallel to the laser propagation as well as the polarization direction. This enables a controllable approach that rotates the trapped particle with fine accuracy and could be used in partial measurement. However, due to the complexity of the interaction of the optical field and refractive index distribution, the trapping trajectory of the Janus particle in the linearly polarized optical trap is still uncovered. In this paper, we focus on the dynamic trapping process and the steady position and orientation of the Janus particle in the optical trap from both simulation and experimental aspects. The trapping process recorded by a high speed camera coincides with the simulation result calculated using the *T*-matrix model, which not only reveals the trapping trajectory, but also provides a practical simulation solution for more complicated structures and trapping motions.

## 1. Introduction

Optical tweezers [1] are a precise tool in micro-manipulating [2,3,4]. With piezo-electric or acoustic-optic devices, optical tweezers easily translate the trapped particle into a 3D space [5]. However, rotation in optical tweezers needs to either modulate the trapping laser [6,7,8,9,10,11,12] or the trapped particles [13,14,15,16,17]. In early research [18], we reported a controllable rotation method based on composite Janus particles together with a linearly polarized optical trap [18]. The spherical Janus particle is chemically synthesized and consists of two hemispheres with different refractive indexes (RI). Janus particles have achieved significant advance over the last decade and are widely applied in drug delivery, micro-manipulating and sensing [19,20,21]. In the linearly polarized trap, a spherical Janus particle tends to align its interface of the two hemispheres parallel to the propagation as well as the polarization direction automatically. By rotating the polarization direction of laser, the particle can rotate about the beam axis. The rotation direction and angular velocity can adjust in real time. The rotation state can be visualized directly using a camera. Considering the mature synthesis technology of Janus particles and the diversity of their structures, the Janus particles must have a promising future in achieving flexible rotation in optical traps and have applications in detecting on a microscale [20,21].

In a previous paper [18], we reported the steady position and orientation of Janus particles in a linearly polarized laser trap using electromagnetic energy theory and an optical tweezers experiment, but the dynamic trapping process was not covered. Involving the relative position of the Janus particle center to the trap center, as well as the relative angle of the two hemispheres interface to the laser propagation or polarization direction, the trapping process of the Janus particle is much more complicated and raises a great challenge for trapping simulations and experiments. The energy theory is powerful in explaining the physical principle of optical trapping, but unhelpful in describing the trapping process. On the other hand, optical force is an indispensable tool in understanding the trapping process, especially in predicting the trapping trajectory [22,23,24,25,26,27]. The geometrical optics model [28,29,30,31] and the electromagnetic scattering model [32,33,34] are two popular models used in calculating the optical force and torque for micro-sized particles. The geometrical optics model is simple and suitable for homogeneous micro-spheres, while the electromagnetic scattering model is outstanding for inhomogeneous particles, such as the Janus particle, as found in our research. The transition matrix (*T*-matrix) method [35,36,37,38,39,40,41,42,43] is widely used in calculating electromagnetic scattering problems by relating the scattering field to the incident field through a transition matrix, which is often denoted as T. The transition matrix *T* depends only on particle’s parameters and is independent of the incident field. Although calculating matrix *T* is complicated, it only needs to be calculated once and can then be used repeatedly, making the *T*-matrix method highly efficient in modelling optical tweezers, when compared to all alternative methods.

This paper focuses on the trapping process of spherical Janus particles in a linearly polarized optical trap. The *T*-matrix method was chosen to simulate the trapping process. Both basic principles of the *T*-matrix method [35] and calculation details are included. The simulation results reveal that the Janus particle always rotates to align its two hemispheres’ interfaces parallel to the laser propagation direction quickly and parallel to the laser polarization direction slowly. The specific trapping process relates to the intensity distribution of a linearly polarized optical trap. The simulated trapping process was verified experimentally with the help of a high speed camera. The simulation and experiment coincided with each other well, which not only describes the trapping process, but also verifies the simulation model and offers an opportunity for studying other, more complicated processes with this model.

## 2. Basic Theory of *T*-Matrix

In the *T*-matrix method, a Gaussian laser beam is expanded through vector spherical wave functions (VSWFs), which is the complete set of the orthogonal basis for solutions of the vector Helmholtz equation in spherical coordinates. The optical trap formed with the Gaussian beam is focused on the optical field physically. The incident field is defined as an optical field in the absence of scattering particles and could be expanded as:

(1) $$E_{incident}=\overset{\infty }{\underset{n=1}{\sum }}\overset{n}{\underset{m=-n}{\sum }}a_{nm}M_{nm}^{\left(1\right)}\left(kr\right)+b_{nm}N_{nm}^{\left(1\right)}\left(kr\right)$$

where $M_{nm}^{\left(1\right)}\left(kr\right)$ and $N_{nm}^{\left(1\right)}\left(kr\right)$ are regular VSWFs for the incident field, so they are singularity-free at the origin. $a_{nm}$ and $b_{nm}$ are expansion coefficients. They are determined by the incident beam parameters and calculated through the integral method [44] or point-matching method [45]. 

Similarly, the scattered field, which is the change of the optical field due to the presence of the particle, can also be expanded in terms of VSWFs:

(2) $$E_{scattered}=\overset{\infty }{\underset{n=1}{\sum }}\overset{n}{\underset{m=-n}{\sum }}p_{nm}M_{nm}^{\left(3\right)}\left(kr\right)+q_{nm}N_{nm}^{\left(3\right)}\left(kr\right)$$

where $M_{nm}^{\left(3\right)}\left(kr\right)$ and $N_{nm}^{\left(3\right)}\left(kr\right)$ are VSWFs for the scattered field. $p_{nm}$ and $q_{nm}$ are expansion coefficients for the scattered field. They are the product of transition matrix *T* and expansion coefficients of the incident field:

(3) $$\left[\begin{array}{c} p_{nm}\\ q_{nm} \end{array}\right]=T\left[\begin{array}{c} a_{nm}\\ b_{nm} \end{array}\right]$$

*T* is the transition matrix of the scattered field to the incident field. Calculating the matrix *T* properly is most significant but challenging, especially for a particle with a complicated structure. However, matrix *T* is independent of the optical field and only determined by the parameters of the scattering particle, including size, shape and refractive index distribution. Even the particle’s position and orientation are coupled to matrix *T* through coordinate transformation rather than recalculation. As a result, calculating matrix *T* is time consuming but needs to be done only once, making the *T*-matrix a tough but efficient simulation method.

In an optical trap, force and torque asserted on a particle are the functions of the momentum transfer from laser to particle, which can be calculated with the expansion coefficients of $a_{nm}$, $b_{nm}$ and $p_{nm}$, $q_{nm}$. Driven by optical force and torque, the particle translates and rotates continually until force and torque go back to zero. With the *T*-matrix method, a particle’s trapping trajectory and steady position and orientation can be simulated through multiple iterations. Additionally, the influence of particle structure on trapping can also be studied.

## 3. Calculating Transition Matrix *T*

### 3.1. Transition Matrix of Homogeneous Particles

For homogeneous microspheres, the transition matrix *T* is a diagonal matrix and the diagonal elements are Mie scattering coefficients. A widely used method is the extended boundary condition method (EBCM) [46], especially for a particle with rotational symmetry. In EBCM, for a homogeneous particle with a radius of *r*, with a RI of $n_{p}$, immersed in a medium with a RI of $n_{m}$, its transition matrix *T* is given as:

(4) $$T=-Q_{p}^{11}\left(k_{m}r,k_{p}r\right){\left[Q_{p}^{31}\left(k_{m}r,k_{p}r\right)\right]}^{-1}$$

where $k_{m}$ and $k_{p}$ are wavenumbers in the medium and particle, respectively. $Q_{p}^{11}$ and $Q_{p}^{31}$ are two partitioned matrices of the same size:

(5) $$Q_{p}^{\mathrm{gh}}\left(k_{m}r,k_{p}r\right)=\left[\begin{array}{cc} {\left(Q_{p}^{\mathrm{gh}}\right)}_{\nu \mu }^{11} & {\left(Q_{p}^{\mathrm{gh}}\right)}_{\nu \mu }^{12}\\ {\left(Q_{p}^{\mathrm{gh}}\right)}_{\nu \mu }^{21} & {\left(Q_{p}^{\mathrm{gh}}\right)}_{\nu \mu }^{22} \end{array}\right]$$

In which the four sub-matrices are integral about the surface enclosing the particle:

(6) $${\left(Q_{p}^{\mathrm{gh}}\right)}_{\nu \mu }^{11}=\frac{jk_{p}{}^{2}}{\pi }\int \left\{\begin{array}{c} \left[n\left(r\right)\times M_{\mu }^{h}\left(k_{p}r\right)\right]\cdot N_{\bar{\nu }}^{g}\left(k_{m}r\right)\\ +\frac{k_{p}}{k_{m}}\left[n\left(r\right)\times N_{\mu }^{h}\left(k_{p}r\right)\right]\cdot M_{\bar{\nu }}^{g}\left(k_{m}r\right) \end{array}\right\}{\mathrm{dS}}_{p}$$

(7) $${\left(Q_{p}^{\mathrm{gh}}\right)}_{\nu \mu }^{12}=\frac{jk_{m}{}^{2}}{\pi }\int \left\{\begin{array}{c} \left[n\left(r\right)\times N_{\mu }^{h}\left(k_{p}r\right)\right]\cdot N_{\bar{\nu }}^{g}\left(k_{m}r\right)\\ +\frac{k_{p}}{k_{m}}\left[n\left(r\right)\times M_{\mu }^{h}\left(k_{p}r\right)\right]\cdot M_{\bar{\nu }}^{g}\left(k_{m}r\right) \end{array}\right\}{\mathrm{dS}}_{p}$$

(8) $${\left(Q_{p}^{\mathrm{gh}}\right)}_{\nu \mu }^{21}=\frac{jk_{m}{}^{2}}{\pi }\int \left\{\begin{array}{c} \left[n\left(r\right)\times M_{\mu }^{h}\left(k_{p}r\right)\right]\cdot M_{\bar{\nu }}^{g}\left(k_{m}r\right)\\ +\frac{k_{p}}{k_{m}}\left[n\left(r\right)\times N_{\mu }^{h}\left(k_{p}r\right)\right]\cdot M_{\bar{\nu }}^{g}\left(k_{m}r\right) \end{array}\right\}{\mathrm{dS}}_{p}$$

(9) $${\left(Q_{p}^{\mathrm{gh}}\right)}_{\nu \mu }^{22}=\frac{jk_{m}{}^{2}}{\pi }\int \left\{\begin{array}{c} \left[n\left(r\right)\times N_{\mu }^{h}\left(k_{p}r\right)\right]\cdot N_{\bar{\nu }}^{g}\left(k_{m}r\right)\\ +\frac{k_{p}}{k_{m}}\left[n\left(r\right)\times M_{\mu }^{h}\left(k_{p}r\right)\right]\cdot N_{\bar{\nu }}^{g}\left(k_{m}r\right) \end{array}\right\}{\mathrm{dS}}_{p}$$

In the four equations above, $S_{p}$ is the surface enclosing the particle. n(r) is the normal vector pointing to the outside of the particle. $M^{h}\left(kr\right)$, $N^{h}\left(kr\right)$ are regular VSWFs when superscript h = 1 or outward VSWFs when superscript h = 3. Subscript μ, ν, $\bar{\nu }$ are composite indices for VSWFs: μ=(m,n), ν=(m,n), $\bar{\nu }=\left(-m,n\right)$.

### 3.2. Transition Matrix of Janus Particles

**Figure 1:**
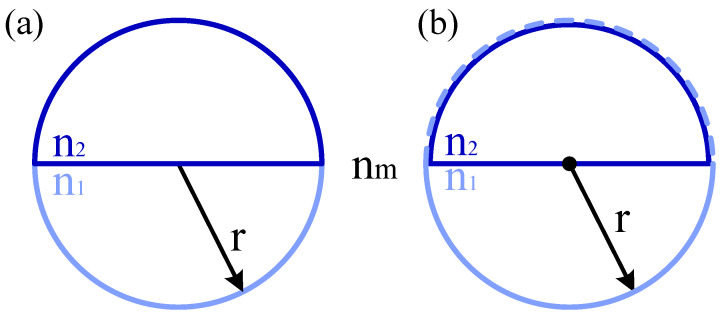
(**a**) Structure and (**b**) calculation model of Janus particles.

The structure of the Janus particle is shown in Figure 1a. It is composed of two hemispheres with different RIs ($n_{1}$ represents the RI of the light blue hemisphere and $n_{2}$ represents the RI of the dark blue hemisphere). The calculation model of Janus particles is shown in Figure 1b. The light blue dash line is a virtual interface, which does not exist except for the purposes of calculation. In this model, we regard the two-hemispheres-shaped Janus particle as a double-layered particle. The outer shell is a homogeneous sphere with a radius of r, with a RI of $n_{1}$, immersed in a medium with a RI of $n_{m}$. The inner core is a homogeneous hemisphere with a radius of r, with a RI of $n_{2}$, immersed in a medium with a RI of $n_{1}$. Since the sphere-shaped shell and the hemisphere-shaped core have the same radius, the origin of the core (the center of the inscribed circle) departs from the center of the sphere by r/2. The transition matrix *T* of the double-layered particle is given as:

(10) $$T=\left(T_{sph}-Q_{sph}^{13}{\tilde{T}}_{h\_sph}Q_{sph}^{31}{}^{-1}\right)\times {\left(I+Q_{sph}^{33}{\tilde{T}}_{h\_sph}Q_{sph}^{31}{}^{-1}\right)}^{-1}$$

In Equation (10), I is an identity matrix. $Q_{sph}^{13}$ $Q_{sph}^{31}$ and $Q_{sph}^{33}$ are the same as in Equation (5) for the sphere-shaped shell, given as: $Q_{sph}^{13}\left(k_{m}r,k_{1}r\right),$ $Q_{sph}^{31}\left(k_{m}r,k_{1}r\right)$ and $Q_{sph}^{33}\left(k_{m}r,k_{1}r\right)$. $T_{sph}$ is the transition matrix of the sphere-shaped shell. According to Equation (4), $T_{sph}$ is given as:

(11) $$T_{sph}=-Q_{\mathrm{sph}}^{11}\left(k_{m}r,k_{1}r\right){\left[Q_{\mathrm{sph}}^{31}\left(k_{m}r,k_{1}r\right)\right]}^{-1}$$

As the origin of core does not overlap with origin of the shell, ${\tilde{T}}_{h\_sph}$ is not the transition matrix of the hemisphere-shaped core, but of the transformed matrix, given as:

(12) $${\tilde{T}}_{h\_sph}=T\left(-\frac{n_{1}r}{2}\right)T_{h\_sph}T\left(\frac{n_{1}r}{2}\right)$$

$T\left(\frac{n_{1}r}{2}\right)$ is the translational matrix. It is worth noting that the translational length is not r/2, but the optical length of $\frac{n_{1}r}{2}$. $T_{h\_sph}$ is the transition matrix of the inner hemisphere. Same with Equation (4), $T_{h\_sph}$ is given as:

(13) $$T_{h\_sph}=-Q_{h\_sph}^{11}\left(k_{1}r,k_{2}r\right){\left[Q_{h\_sph}^{31}\left(k_{1}r,k_{2}r\right)\right]}^{-1}$$

## 4. Simulation Results

### 4.1. Coordinate Description of the Janus Particle

**Figure 2:**
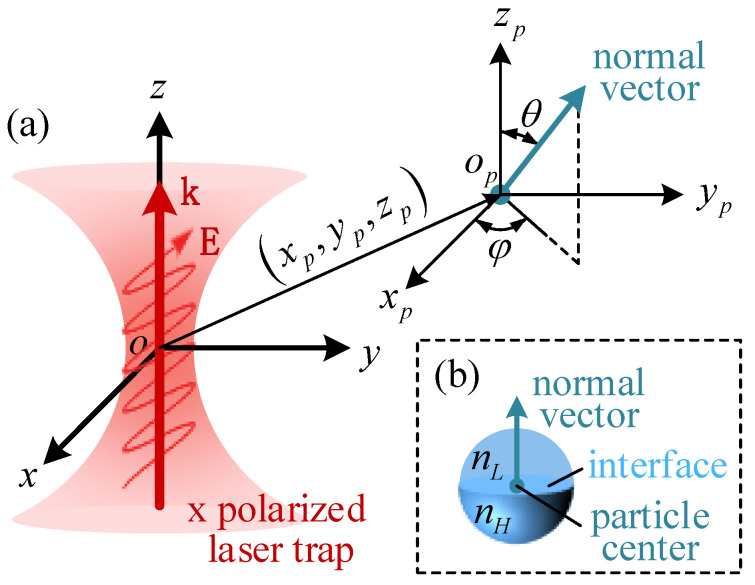
(**a**) The coordinate system to describe the Janus particle’s position and orientation; (**b**) the schematic diagram of the Janus particle’s structure and the virtual normal vector.

The Janus particle is a spherical particle composed of two hemispheres, so it has five degrees of freedom (DOFs): three translational DOFs and two rotational DOFs. To describe the Janus particle’s position and orientation quantificationally, we introduce two coordinate systems and a virtual normal vector in this paper. As is depicted in Figure 2a, the global coordinate system *oxyz* is located at the trap center. Laser propagation direction and polarization direction are defined as *z* direction and x direction, respectively. The local coordinate system, $o_{p}x_{p}y_{p}z_{p}$, is located at the particle center. All axes in $o_{p}x_{p}y_{p}z_{p}$ are parallel to the axes in *oxyz*. The virtual normal vector is located at the Janus particle’s center and points from the hemisphere with a high RI to the hemisphere with a lower RI (Figure 2b). The three translational DOFs are described by the particle’s center in the *oxyz* system. The two rotational DOFs are described by the pitch angle θ and azimuth angle φ of the normal vector in the $o_{p}x_{p}y_{p}z_{p}$ system (the pitch angle *θ* is the angle from $z_{p}+$ axis to the normal vector, 0 ≤ *θ* ≤ π. The azimuth angle *φ* is the angle from $x_{p}+$ axis to the projection of the normal vector in the $o_{p}x_{p}y_{p}$ plane, 0 ≤ *φ* ≤ 2π).

### 4.2. Trapping Trajectory and Steady Position and Orentation of Janus Particles

**Figure 3:**
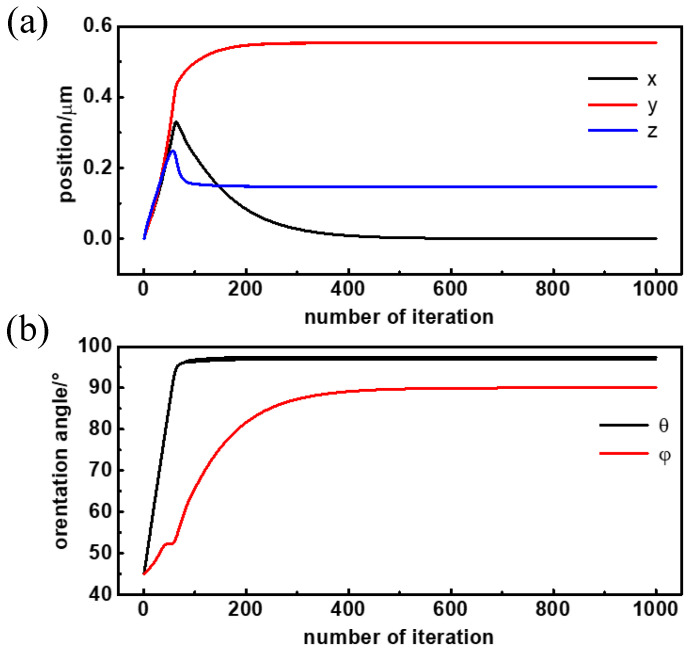
(**a**) Translational and (**b**) rotational trajectory of the Janus particle in the linearly polarized optical trap.

This section focuses on the simulated translational and rotational trajectory of the Janus particle in a linearly polarized optical trap. To match the simulation parameters to the real Janus particles in the following trapping experiment, the radius of the Janus particle was set as 2 μm, and the RIs of the two hemispheres were set as 1.57 and 1.49 respectively. The particle was immersed in an aqueous environment with a RI of 1.33. The initial position of the Janus particle was set as (0, 0, 0), meaning that the particle center was at the trap center. The initial orientation was *θ* = 45°, *φ* = 45°, meaning that the angle between laser propagation direction (*z*-direction) and virtual normal vector was set as 45°, while the angle between the laser polarization direction (*x*-direction) and the projection of the virtual normal vector was also 45°. The simulated translational and rotational trajectory is shown in Figure 3a,b, respectively. Since the step size of Janus particle’s position and orientation is linearly related to optical force and torque during the simulation, we used the iteration number to represent real time in the following simulation curve.

The simulated steady position and orientation of the Janus particle in an x-polarized laser trap is (*x*, *y*, *z*, *θ*, *φ*) = (0, 0.55 μm, 0.15 μm, 97.2°, 90°). As published in our previous paper, the Janus particle’s interface of two hemispheres was almost parallel to the laser propagation direction (*θ* = 97.2°) and was strictly parallel to the polarization direction (*φ* = 90°) when it is stable. What is more, the trap center did not overlap with the particle center but was located in the hemisphere with the higher RI (*y* = 0.55 μm). The specific position and orientation are the result of the interaction of the laser trap’s intensity distribution and the Janus particle’s RI distribution. Since the interface of the two hemispheres is always parallel to the laser polarization, adjusting the polarization direction can induce the trapped Janus particle to rotate about the *z*-axis synchronously, which is a controllable rotating method based on laser and the particle’s anisotropy. 

Apart from the steady position and orientation, it is worth noting that the pitch angle *θ* reaches the stable value fast, while the azimuth angle *φ* reaches the stable value slowly. In Figure 2b, the pitch angle *θ* iterates 100 times for a rotation angle of 52.2° (97.2° − 45° = 52.2°) to the final stable state, while the azimuth angle *φ* iterates more than 500 times for a rotation angle of 45° (90° − 45° = 45°) to the final stable orientation. This means the Janus particle rotates to align to the interface of two hemispheres parallel to the laser propagation direction preferentially, but parallel to laser polarization direction secondarily. 

This weird phenomenon results from the specific intensity distribution of linearly polarized optical traps. The optical intensity extends the most in the propagation direction (*z*-direction), then the polarization direction (*x*-direction), and finally in the y-direction. According to the electromagnetic energy theory, when a particle reaches its steady position and orientation, the electromagnetic energy must be lowest, which means the particle hemisphere of the highest RI overlaps with the optical field of highest intensity as much as possible. So, the Janus particle moves in the trap continuously to locate the hemisphere with a higher RI at the trap center (*y* = 0.55 μm) and, at the same time, rotates to align the interface of the two hemispheres parallel to the laser propagation (*θ* = 97.2°) direction approximately and parallel to polarization direction (*φ* = 90°) strictly. The translational and rotational velocity is determined by the derivative of the electromagnetic energy. The optical intensity distribution in the longitudinal direction (*z*-direction) is much larger than in the transversal direction (*x*- and *y*-direction), while the intensity in the polarization direction (*x*-direction) is just slightly larger than in the y-direction. So, the torque in the pitch direction, which is related to the intensity difference in the longitudinal and transversal directions, is much larger than in the azimuth direction, which is related to the intensity difference in the polarization and y-direction. As a result, the Janus particle aligns the interface of its two hemispheres parallel to the propagation direction first and quickly (*θ* = 97.2°), and then parallel to the polarization direction slowly (*φ* = 90°). 

### 4.3. Trapping Process of Janus Particle with a Curved Interface

**Figure 4:**
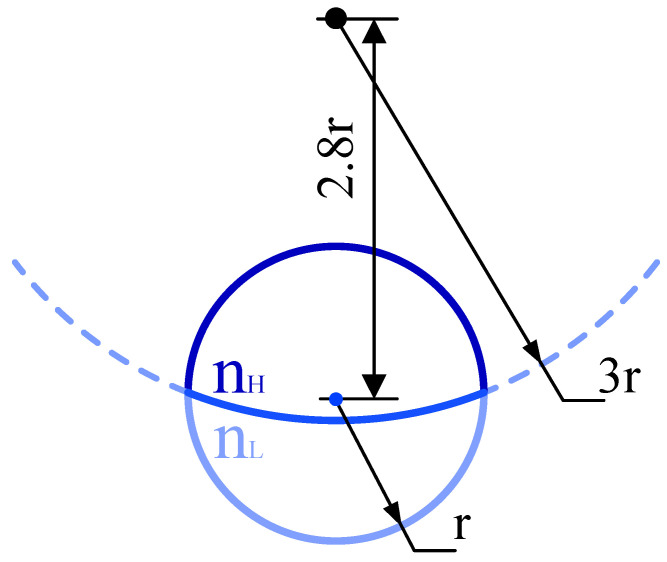
Simulation model of the Janus particle with a curved interface.

The simulation above is based on the model of the Janus particle with a plane interface between the two hemispheres. However, due to the difference in surface tension between materials, the interface of the two hemispheres is actually curved. Figure 4 shows the simulation model of the Janus particle with the curved interface. The radius of the interface between the two hemispheres is three times that of the radius of the Janus particle. To make sure that the volumes of the two hemispheres are equal, the deflection between the interface center and the particle center is 2.8r (r is the radius of the Janus particle).

The simulation condition and coordinate description are exactly the same as in Section 4.2. RIs of the two hemispheres are 1.57 and 1.49 respectively. The RI of the environment is 1.33. The particle’s radius is 2 μm. The Janus particle’s position and orientation are also described by (*x*, *y*, *z*) and (*θ*, *φ*). (*x*, *y*, *z*) describe the position of the Janus particle’s center relative to the trap center. The pitch angle *θ* and the azimuth angle *φ* are also defined by a virtual normal vector and describe the Janus particle’s orientation relative to the laser propagation and polarization direction. Since the interface of the two hemispheres is circular, the virtual normal vector is defined as the normal vector of the circular interface, which is located at the Janus particle’s center and points to the hemisphere with the lower RI. It is worth noting that the circular interface cannot be parallelly aligned to any direction strictly, but the tangent plane of the normal vector does align parallelly to the propagation direction and the polarization direction when (*θ*, *φ*) = (90°, 90°). For a coherent account of the plane interface, (*θ*, *φ*) = (90°, 90°) is explained as the interface of the two hemispheres (actually the tangent plane of circular interface) parallel to the propagation and polarization direction.

**Figure 5:**
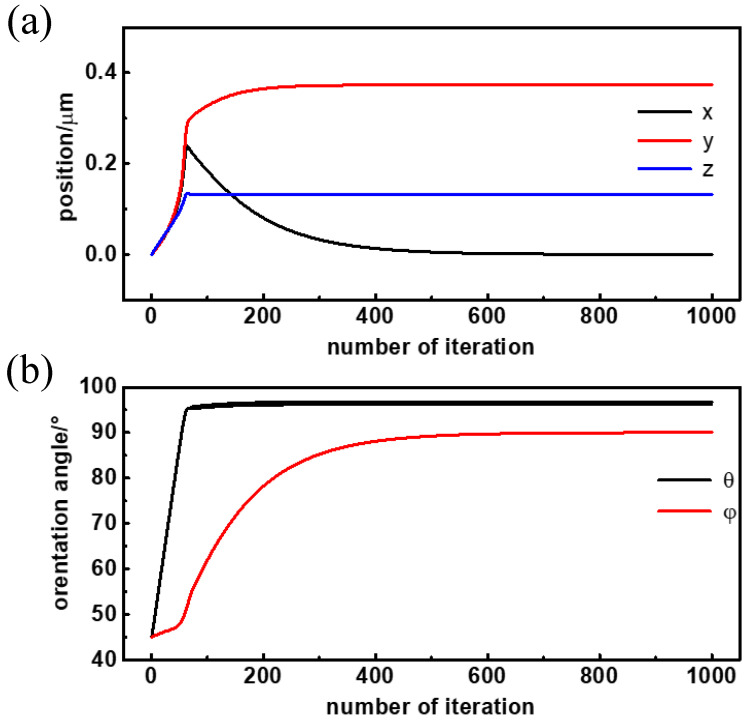
(**a**) Translational and (**b**) rotational trajectory of the Janus particle with a curved interface.

The Janus particle’s initial position and orientation is (*x*, *y*, *z*, *θ*, *φ*) = (0, 0, 0, 45°, 45°). The trapping trajectory in the x-polarized optical trap is shown in Figure 5. The steady position and orientation is (*x*, *y*, *z*, *θ*, *φ*) = (0, 0.373, 0.132, 96.8°, 90°), very similar to the steady position and orientation of the particle with the plane interface. The simulation result reveals that the curvature of the interface only changes the deflection of the Janus particle’s center to the trap center but has no influence on particle’s orientation. The interface of the two hemispheres is still parallel to the laser propagation direction, approximately (*θ* = 96.8°), and parallel to the polarization direction strictly, (*φ* = 90°). What is more, the azimuth angle φ takes a longer time to reach the steady position and orientation than the pitch angle *θ*, which is consistent with the simulation result of the Janus particle with a plane interface.

## 5. Experiment Results

**Figure 6:**
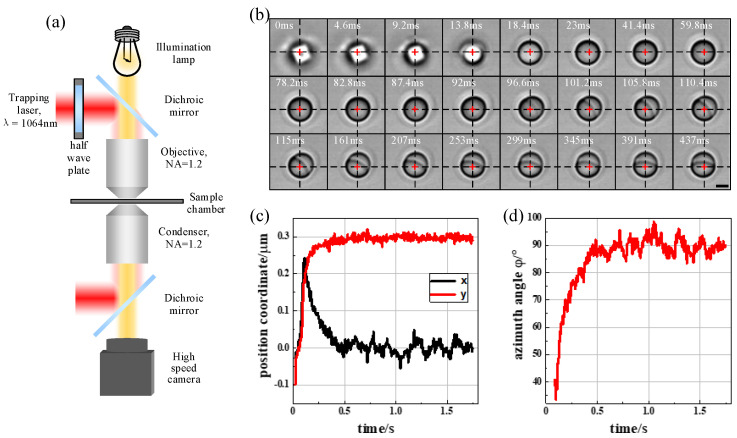
(**a**) Schematic of the optical trapping setup. (**b**) Trapping process of the Janus particle in the x-polarized optical trap as recorded by a high speed camera. The darker hemisphere represents the PS hemisphere, while the lighter hemisphere represents the PMMA hemisphere due to their RI difference. Red crosses represent the trap center. Scale bar 2 μm. (**c**) Extracted position and (**d**) orientation of the Janus particle versus trapping time.

To demonstrate our simulation, we used optical tweezers together with a high speed camera to record the trapping process of the Janus particle in a linearly polarized laser trap. The schematics of the optical tweezers’ setup is shown in Figure 6a. A linearly polarized laser with wavelength of 1064 nm was focused by a high numerical aperture (NA = 1.2) with the objective to form the optical trap. The polarization direction of the trap was controlled by an electric motor-driven half wave plate (HWP). A sample chamber was placed in the focal plane of objective and contained the aqueous solution of Janus particles. Particles near the trap center were able to be captured by the optical trap automatically in less than one second. A camera with acquisition frequency of 217 Hz was placed in the conjugate plane of the trap center and recorded the trapping process. 

The Janus particle used in this trapping experiment was synthesized with the microfluidic method [19, 20, 21]. The two hemispheres of the Janus particle were made of polystyrene (PS) and polymethyl-methacrylate (PMMA), respectively. Since the RI of the PS, which was 1.57, was slightly larger than the RI of the PMMA, which was 1.49, the PS-hemisphere showed a darker gray color than the PMMA-hemisphere in the recorded images (Figure 6b), providing a directional method to detect the particle’s orientation. The average radius of the Janus particle was about 2 μm. During the trapping experiment, the Janus particles were suspended in sodium dodecyl sulfonate (SDS) aqueous solution to avoid aggregating. The radius of the Janus particle, the RI of the two hemispheres and the medium were all the same for the simulation model and real particles (*r* = 2 μm, $n_{L}$ = 1.49, $n_{H}$ = 1.57,$\;n_{m}$ = 1.33).

Since the initial position and orientation of the Janus particle was random and difficult to control, we recorded multiple trapping processes of the Janus particle from a free state to a stably trapped state. All experiment results show that the interface of the two hemispheres rotated to align parallelly with the propagation direction extremely quickly and then rotated to align parallelly to the polarization direction slowly. The experiment results coincide well with the simulation results. To further compare the simulation and experiment results, a specific trapping process of the Janus particle in a horizontally polarized (x-polarized) optical trap is shown in Figure 6b. Every image has been marked with time (white text) and the trap center position (red cross). The minimum time interval is 4.6 ms for sequential images. To reveal the complete trapping process and highlight the rotation details, the time interval between adjacent images is not the same.

In Figure 6b, from t = 0 ms to t = 13.8 ms, the images are totally out of focus, meaning that the particle was not trapped at all. From t = 18.4 ms to t = 87.4 ms, the images are focused, indicating the particle was trapped. However, the recorded images show homogeneous particles, meaning the interface of the two hemispheres was perpendicular to the laser propagation direction at that moment. When t = 92 ms, the PMMA hemisphere started to emerge in the upper right corner of the particle. When t = 96.6 ms, The PMMA hemisphere became a little clearer. After t = 101.2 ms, the PMMA hemisphere (the light gray hemisphere) and the PS hemisphere (the dark gray hemisphere) were both clear enough, meaning that the interface of the two hemispheres had already rotated to be parallel with the laser propagation direction. From t = 105.8 ms to t = 437 ms, the interface of the two hemispheres rotated in an oxy plane and was finally parallel to the polarization direction (*x*-axis). In this experiment, the Janus particle spent less than 101.2 ms to align the interface parallelly to the propagation direction, while it spent about 437 ms to align parallelly to the polarization direction. So, it takes a longer time to rotate the interface of the two hemispheres parallel to the polarization direction, which coincides with the simulation results above.

The *x*-coordinate, *y*-coordinate and azimuth angle *φ* in Figure 6b were extracted using through image processing (the *z*-coordinate and pitch angle *θ* could not obtain an exact value using the present system). The Janus particle’s position and orientation versus its trapping time are shown in Figure 6c,d, respectively. The trapping curves show a similar trajectory to the simulation results in Figure 3 and Figure 5. The steady position and orientation of the Janus particle in the trapping experiments, together with simulation results, are listed in Table 1. Experiment results coincide well with the simulation results, especially more closely to the simulation model of the curved interface

## 6. Discussion

This paper focuses on the trapping trajectory and steady position and orientation of the Janus particle in a linearly polarized optical trap in both simulation and experiment aspects. Since the Janus particle is composed of two hemispheres with different refractive indexes, it has a more complicate structure than the homogeneous particle. A new calculation model was built to simulate the trapping trajectory of Janus particles. Simulation results revealed that, no matter what the curvature of the interface was, trap center was always located in the hemisphere with a high RI. What is more, the interface of the two hemispheres was always approximately parallel to the laser propagation direction and strictly parallel to the polarization direction independent of curvature. This follows the lowest electromagnetic energy principle. The curvature may change the deflection between particle’s center to the trap center but does not influence the particle’s orientation relative to the polarization direction. This specific property provides a practical approach of controllable rotation based on the Janus particles in linearly polarized optical traps. The simulation also draws the trapping trajectory clearly. The Janus particle’s interface of two hemispheres always rotates to be parallel to the propagation direction fast and quickly and rotates to be parallel to the polarization direction slowly. The simulated trapping trajectory was verified by the optical trapping experiment of real PS/PMMA composite particles with the help of a high speed camera. The optical trapping experiment coincided with simulation result very well, which not only explained the trapping trajectory of the Janus particle, but also confirmed the simulation model and provided a practical method for calculating more complicated structures and trapping trajectories. Moreover, the simulation model builds a relationship between the parameters of the Janus particle structure to its trapping trajectory, so it is a powerful tool in designing the particle’s structure to realize a specific trapping property.

## Figures and Tables

**Table 1:** Steady position and orientation of the Janus particle in the simulation and experiment.

	*x*/μm	*y*/μm	*z*/μm	*θ*	*φ*
Simulation of plane interface	0	0.553	0.147	97.7°	90°
Simulation of curved interface	0	0.373	0.132	96.8°	90°
Experiment with real particles	−0.001	0.298	-	-	89.88°

## Data Availability

Not applicable.
